# The induction of the transcription factor Nrf2 enhances the antinociceptive effects of delta-opioid receptors in diabetic mice

**DOI:** 10.1371/journal.pone.0180998

**Published:** 2017-07-10

**Authors:** Christina McDonnell, Sergi Leánez, Olga Pol

**Affiliations:** 1 Grup de Neurofarmacologia Molecular, Institut d’Investigació Biomèdica Sant Pau, Barcelona, Spain; 2 Institut de Neurociències, Universitat Autònoma de Barcelona, Barcelona, Spain; University of Edinburgh, UNITED KINGDOM

## Abstract

The involvement of heme oxygenase 1 (HO-1) in the modulation of the antinociceptive effects of opioids in type 1 diabetes has been demonstrated but the role played by the transcription factor Nrf2 in the regulation of painful neuropathy and in the effects and expression of δ-opioid receptors (DOR) in type 2 diabetes, has not been studied. In male BKS.Cg-m+/+Leprdb/J (db/db) mice, the anti-allodynic effects produced by a Nrf2 transcription factor activator, sulforaphane (SFN) administered alone and combined with two DOR agonists, [d-Pen(2),d-Pen(5)]-Enkephalin (DPDPE) and (+)-4-[(αR)-α-((2S,5R)-4-Allyl-2,5-dimethyl-1-piperazinyl)-3-methoxybenzyl]-N,N diethylbenzamide (SNC-80), were evaluated. The effects of SFN on glucose levels and body weight as well as on the proteins levels of Nrf2, HO-1, NAD(P)H: quinone oxidoreductase 1 (NQO1), MAPKs (JNK) and DOR in sciatic nerve from db/db mice were also assessed. This study showed that the administration of SFN dose dependently reversed mechanical allodynia, reduced hyperglycemia and body weight gain associated to type 2 diabetes and significantly increased the anti-allodynic effects of DPDPE and SNC-80 in db/db mice. This treatment normalized the down regulation of Nrf2 and NQO1 and enhanced the protein levels of HO-1 in db/db mice. Moreover, the administration of SFN also inhibited the JNK phosphorylation and DOR down-regulation in the sciatic nerve of diabetic mice. Our data indicated that SFN treatment is effective in reversing mechanical allodynia and enhancing DOR antinociceptive effects in db/db mice which effects might be mediated by activating Nrf2 signaling, reducing hyperglycemia, inhibiting JNK phosphorylation and avoiding DOR down-regulation in the sciatic nerve of these animals. These results propose SFN, alone and/or combined with DOR agonists, as interesting approaches for the treatment of painful diabetic neuropathy associated to type 2 diabetes in mice.

## Introduction

Diabetic neuropathy and oxidative stress are two of the major complications associated with the development of diabetes that affects between 40–50% of people who suffer from this disorder. The clinical characteristics of diabetic neuropathy ranging from sensory deficit to allodynia (painful reaction to innocuous stimuli), and an increased sensitivity to painful stimulus [1, 2]. However, despite being one of the major symptoms of this metabolic disorder, diabetic neuropathy remains difficult to treat which highlight the importance of finding new therapeutic strategies.

Hyperglycemia is one of the principal mechanisms involved in the development of diabetes and in the numerous complications associated with this disease including diabetic neuropathy [2–4]. Therefore, high glucose levels by increasing the production of reactive oxygen species (ROS) and inflammatory mediators are prominent contributors to nerve dysfunction and the subsequent pain observed in diabetic patients. The induction of ROS is a characteristic of oxidative stress, and insulin can promote its elimination by regulating the production of several intracellular antioxidants in a Nrf2 transcription factor dependent pathway [5]. This transcription factor, in addition to modulating the expression of numerous genes which control the immune and inflammatory responses [6], also regulates the expression of several genes which control antioxidant and detoxifying enzymes, such us the heme oxygenase 1 (HO-1) and NAD(P)H quinone oxidoreductase 1 (NQO1). The overexpression of these enzymes inhibited several neuro-inflammatory responses responsible for the induction of diabetic neuropathy, revealing the important modulatory role played by the activation of Nrf2 signaling pathway against oxidative stress and the inflammatory cascade associated to diabetes [6].

It is well known that the over-expression of HO-1 is associated with potent anti-inflammatory and antinociceptive effects [7, 8]. Indeed, the administration of HO-1 inducer compounds, such as cobalt protoporphyrin IX (CoPP) inhibits acute thermal nociception [9], inflammatory [10–12], visceral [11] as well as neuropathic pain induced by nerve injury, vincristine injection, or associated with type 1 diabetes in rodents [8, 13, 14]. Nevertheless, the possible antinociceptive effects produced by the activation of the Nrf2 signaling pathway on the mechanical allodynia associated with type 2 diabetes in mice have not been studied.

Mitogen activated protein kinases (MAPK) are a group of intracellular messenger proteins that transmit signals from cell membranes to receptors to the nucleus. The MAPK family consists of extracellular signal-regulated protein kinases (ERKs), p38 kinases, and c-Jun NH2-terminal kinases (JNK). It is well known that MAPKs play a critical role in the etiology of diabetic neuropathy [15] and several authors have demonstrated that the activation of ERKs and p38 is involved in the development of the mechanical allodynia observed in the early stages of db/db mice [16, 17]. However, little is known about the role played by JNK in the early stages of diabetes in this animal model as well as to the effects of SFN treatment on its expression.

Several studies have shown that diabetic painful neuropathy is difficult to treat due to its resistance to opioids, in particular to μ-opioid receptor (MOR) agonists [18, 19]. In contrast to MOR agonists, some studies have demonstrated the potential antinociceptive effects produced by δ-opioid receptor (DOR) agonists in diabetic animals. Indeed, the intracerebroventricular, intrathecal and systemic administration of DOR agonists inhibited the nociceptive responses associated to type 1 diabetes in rodents [20–22]. A recent study has been also demonstrated that the antinociceptive effects produced by DOR agonists in streptozotocin-induced type 1 diabetes in mice were significantly increased by its co-treatment with an HO-1 inducer compound [22]. Nonetheless, the role played by the systemic administration of DOR agonists on the mechanical allodynia observed in db/db mice and the effects produced by the Nrf2 transcription factor inducer (SFN) on the antinociceptive effects of DOR agonists and its expression in type 2 diabetic animals has not been assessed.

Therefore, in order to evaluate the possible analgesic effects of SFN and DOR agonists, administered alone or in combination in type 2 diabetes neuropathy, in this study we used a well characterized model of diabetes, db/db mice, which exhibit characteristics of type 2 diabetes, including hyperglycemia, obesity as well as mechanical allodynia from 7–12 weeks of age [23–26]. In these animals we evaluated: (1) whether administration of a well-established transcription factor Nrf2 activator (SFN) might effectively attenuates the mechanical allodynia associated with diabetes; (2) the effects of SFN on hyperglycemia and body weight; (3) the antinociceptive effects produced by the subcutaneous administration of two specific DOR agonists ([d-Pen(2),d-Pen(5)]-Enkephalin; DPDPE) and (+)-4-[(αR)-α-((2S,5R)-4-Allyl-2,5-dimethyl-1-piperazinyl)-3-methoxybenzyl]-N,N diethylbenzamide (SNC-80) alone and combined with SFN; (4) the reversibility of DPDPE and SNC-80 antinociceptive effects by their co-administration with a specific DOR antagonist, naltrindole; (5) the effects of SFN treatment on the proteins levels of Nrf2, HO-1, NQO1, JNK and DOR expression in the sciatic nerve of diabetic animals.

## Material and methods

### Animals

The experiments were performed in 7 weeks old male type 2 diabetic (BKS.Cg-m+/+Leprdb/J; db/db) mice and heterozygotes as control (db/+) mice acquired from Charles River Laboratories (France). Animals were housed under 12/12-h light/dark conditions in a room with controlled temperature (22°C) and humidity (66%). Animals had free access to food and water and were used after 6 days of acclimatization to housing conditions. All experiments were conducted between 9:00 AM and 5:00 PM. All efforts were made to minimize animal suffering and to reduce the number of animals used.

All experimental procedures within this study were carried out in accordance with the recommendations in the Guide for the Care and Use of Laboratory Animals of the National Institutes of Health. The protocol was approved by the local Ethical Committee of our Institution (Comissió d’Etica en l’Experimentació Animal i Humana de la Universitat Autònoma de Barcelona). This study was carried out in strict accordance with Universitat Autònoma de Barcelona (Permit Number: 6266).

Mice were monitored daily for general health and well-being and the body weight, abnormal postures, appearance of skin and hair, and behavior were evaluated.

### Nociceptive behavioral tests

Mechanical allodynia was quantified by measuring the hind paw withdrawal response to von Frey filament stimulation. In brief, animals were placed in methacrylate cylinders (20 cm high, 9 cm diameter; Servei Estació, Barcelona, Spain) with a wire grid bottom through which the von Frey filaments (North Coast Medical, Inc., San Jose, CA) with a bending force in the range of 0.008–3.5 g were applied by using a modified version of the up–down paradigm, as previously reported by Chaplan et al. (1994) [27]. The filament of 3.0 g was used as a cut-off. Then, the strength of the next filament was decreased or increased according to the response. The threshold of response was calculated from the sequence of filament strength used during the up–down procedure by using an Excel program (Microsoft Iberia SRL, Barcelona, Spain) that includes curve fitting of the data. Clear paw withdrawal, shaking, or licking of the paw was considered as a nociceptive-like response. Both hind paws were tested. Animals were allowed to habituate for 1 h before testing in order to allow an appropriate behavioral immobility.

### Western blot analysis

Control and db/db mice treated with SFN were killed by cervical dislocation and tissues from sciatic nerves were removed immediately after sacrifice, frozen in liquid nitrogen, and stored at -80°C until assay. Samples of sciatic nerves from two animals were pooled into one experimental sample to obtain enough protein levels for performing the Western blot analysis. The Nrf2, HO-1, NQO1, total JNK, phosphorylated JNK and DOR protein levels were analyzed.

Tissues were homogenized in ice-cold lysis buffer (50 mM Tris·Base, 150 nM NaCl, 1% NP-40, 2 mM EDTA, 1 mM phenylmethylsulfonyl fluoride, 0.5 Triton X-100, 0.1% sodium dodecyl sulfate, 1 mM Na3VO4, 25 mM NaF, 0.5% protease inhibitor cocktail, and 1% phosphatase inhibitor cocktail). All reagents were purchased from Sigma (St. Louis, MO) with the exception of NP-40 from Calbiochem (Darmstadt, Germany). The crude homogenate was solubilized for 1 h at 4°C, sonicated for 10 s and centrifuged at 4°C for 15 min at 700 g. The supernatant (60 μg of total protein) was mixed with 4 x laemmli loading buffer and then loaded onto 4% stacking/10% separating sodium dodecyl sulfate polyacrylamide gels. The proteins were electrophoretically transferred onto PVDF membrane for 120 min, blocked with TBST + 5% nonfat dry milk or BSA and subsequently incubated overnight at 4°C with polyclonal rabbit anti-Nrf2 (1:200, Abcam, Cambridge, United Kingdom); anti-HO-1(1:400, Stressgen, Ann Arbor, MI), anti-NQO1 (1:400, Sigma, St. Louis, MO), anti-phosphorylated JNK (1; 200, Cell Signaling Technology (Danvers, MA, USA), anti-total JNK (1; 200, Cell Signaling Technology (Danvers, MA, USA) and anti-DOR (1:1000, Chemicon-Millipore) antibodies.

The proteins were detected by a horseradish peroxidase-conjugated anti-rabbit secondary antibody (GE Healthcare, Little Chalfont, Buckinghamshire, United Kingdom) and visualized with chemiluminescence reagents (ECL kit; GE Healthcare) and by exposure onto hyperfilm (GE Healthcare). The intensity of blots was quantified by densitometry. The membranes were stripped and re-probed with a monoclonal rabbit anti-β-actin antibody (1:5000, Abcam, Cambridge, United Kingdom) used as a loading control.

### Experimental design

Body weight and glucose levels from the tail blood were measured. One drop of tail blood was analyzed using a glucometer (OneTouch^®^ UltraMini^®^). Blood glucose and body weight were measured at 0, 7 and 11 days after SFN or vehicle treatment. For measuring mechanical allodynia, animals were habituated for 1 h to von Frey filaments test during 4 days. After the habituation period, baseline measurements were established (n = 6 animals per group). Animals were tested at 0, 1, 3, 5, 7, 9 and 11 days after SFN or vehicle treatment.

We evaluated the effects on body weight, glucose levels and mechanical allodynia of the subcutaneous administration of 2.5, 5 and 10 mg/kg of SFN compared to vehicle (n = 6 animals per dose). In these experiments, db/+ mice treated with an equal volume of vehicle were used as controls (n = 6 animals per group). The doses of SFN were selected in accordance to previous pilot studies and other works [28].

In a second set of experiments, we evaluated the mechanical anti-allodynic effects of the subcutaneous administration of different doses of DPDPE and SNC-80 (0.15, 0.5, 1 and 5 mg/kg) compared to vehicle in db/db mice (n = 6 animals per dose) and the reversibility of its effects by the co-administration of 2 mg/kg of naltrindole, a specific DOR antagonist, with a high dose (5 mg/kg) of DPDPE or SNC-80 in db/db mice. The doses of DPDPE and SNC-80 were chosen from the dose-response curves studies, as the ones that produced a maximal antinociceptive effect.

In a third set of experiments, the anti-allodynic effects produced by the intraperitoneal administration of 10 mg/kg of SFN alone or combined with the subcutaneous administration of 0.15 mg/kg of DPDPE and with 0.5 mg/kg of SNC-80 in db/db mice were evaluated (n = 6 animals per group). The doses of DPDPE and SNC-80 were chosen from the dose-response curves performed in this study, as the ones that produced a reduced antinociceptive effect.

Finally, on day 11 of treatment with 10 mg/kg of SFN or vehicle, mice were sacrificed and tissues removed, frozen and preserved for western blot assays to evaluate the protein levels of Nrf2, HO-1 and NQO1, total and phosphorylated JNK and DOR in sciatic nerves from db/db mice. In these experiments, db/+ mice treated with vehicle have been used as controls (n = 4 samples per group).

### Drugs

SFN was acquired from Merck Chemicals and Life Science S.A.U. (Madrid, Spain), dissolved in dimethyl sulfoxide (DMSO; 1% solution in saline) and administered intraperitoneally 2–3 hours before behavioral testing. DPDPE, SNC-80 and naltrindole were purchased from Sigma-Aldrich (St. Louis, MO). DPDPE was dissolved in saline solution (0.9% NaCl) and SNC-80 and naltrindole were dissolved in DMSO (1% solution in saline). DPDPE and SNC-80 were administered subcutaneously and naltrindole intraperitoneally, 30 min before behavioral testing. All compounds were freshly prepared before use and administered in a final volume of 10 ml/kg. Control animals received the same volume of vehicle.

### Statistical analysis

The statistical analysis was performed using the “Statistical Package for Social Sciences” (SPSS, version 17 for Windows, IBM España, Madrid, Spain). Data are expressed as mean ± standard error of the mean (SEM). The initial comparison of glucose levels, body weight and mechanical allodynia between db/db and db/+ mice was evaluated by an unpaired Student’s t test. The effects produced by continuous administration of several doses of SFN on mechanical allodynia, glucose levels and body weight in db/db versus db/+ mice treated with vehicle were evaluated by a two-way ANOVA repeated measures (treatment and days of administration as between factors of variation) followed by the corresponding one-way ANOVA and Student Newman Keuls test.

The comparison of the anti-allodynic effects produced by the subcutaneous administration of different doses of DPDPE, SNC-80 or vehicle was evaluated by using a one way ANOVA followed by the Student Newman Keuls test. The reversal of the antinociceptive effects produced by DPDPE or SNC-80 with naltrindole was also analyzed using a one way ANOVA followed by the Student Newman Keuls test. The comparison of the effects produced by the administration of SFN on the anti-allodynic effects of DPDPE and SNC-80 was also assessed by using a one way ANOVA followed by the Student Newman Keuls test. In these experiments, antinociception in von Frey filaments is expressed as the percentage of maximal possible effect, where the test latencies pre (baseline) and postdrug administration are compared and calculated according to the following equation:

Maximal possible effect (%) = [(drug−baseline)/(cut-off−baseline)] x 100

Changes in the protein levels of Nrf2, HO-1, NQO1, JNK and DOR in the sciatic nerve from db/db mice treated with SFN or vehicle versus db/+ mice treated with vehicle were analyzed by a one-way ANOVA followed by the Student Newman Keuls test. A value of *p <* 0.05 was considered significant.

## Results

### Diabetic neuropathy

In accordance to other reports, higher levels of glucose in db/db mice (492.8 ± 14.7 mg/dL) versus db/+ mice (176.3 ± 7.8 mg/dL; *p <* 0.001; unpaired Student t test; n = 6 animals per group), increased body weight (39.8 ± 0.8 g) in db/db mice versus (21.4 ± 0.3 g) in db/+ mice were observed (*p <* 0.01; unpaired Student t test; n = 6 animals per group). Furthermore, mechanical allodynia was also demonstrated in db/db mice. Indeed, a significant decrease of the threshold for evoking paw withdrawal to a mechanical stimulus in the hind paws of db/db mice (1.7 ± 0.1 von Frey filament strength, g) versus db/+ mice (2.7 ± 0.1 von Frey filament strength, g; *p <* 0.001; unpaired Student’s t test; n = 6 animals per group) was demonstrated.

### Effect of SFN treatment on mechanical allodynia

**Fig 1:**
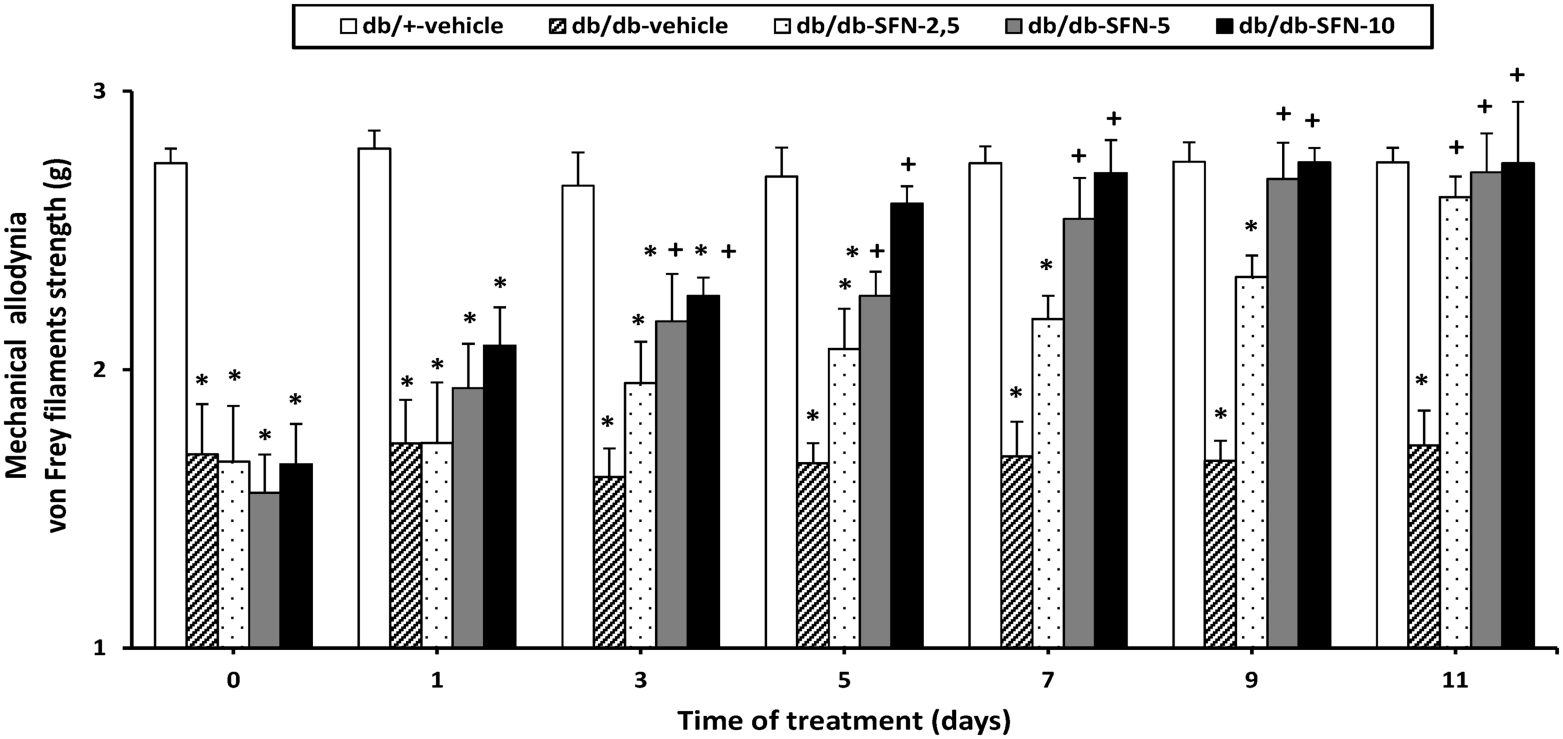
The anti-allodynic effects produced by the intraperitoneal administration of SFN in db/db mice. Mechanical allodynia in the hind paws of db/db mice intraperitoneally treated with vehicle or SFN (2.5, 5 and 10 mg/kg) during 11 consecutive days is shown. The effects of vehicle in db/+ mice are also represented. Data are shown at day 0, 1, 3, 5, 7, 9 and 11 of SFN treatment and expressed as von Frey filaments strength (g). For each day evaluated, * indicates significant differences versus db/+ mice treated with vehicle (*p <* 0.05, one-way ANOVA followed by the Student Newman Keuls test) and + indicates significant differences versus db/db mice treated with vehicle (*p <* 0.05, one-way ANOVA followed by the Student Newman Keuls test). Results are shown as mean values ± SEM; n = 6 animals per experimental group.

The effects of intraperitoneal administration of different doses (2.5, 5 and 10 mg/kg) of SFN during 11 consecutive days on the mechanical allodynia in db/db mice were also studied. On days 0, 1, 3, 5, 7, 9 and 11 of treatment with SFN (Fig 1) the nociceptive responses were assessed. The two way ANOVA repeated measures revealed a significant effect of the treatment (*p <* 0.001), days of administration (*p <* 0.001) and their interaction (*p <* 0.001). That is, the significant decrease of the threshold for evoking hind paw withdrawal to a mechanical stimulus observed in db/db mice treated with vehicle was normalized in animals treated with SFN during 11 consecutive days (*p <* 0.001; one-way ANOVA versus db/db mice treated with vehicle). Moreover, while the mechanical allodynia observed in db/db mice was attenuated on day 5 of treatment with 10 mg/kg of SFN, lower doses of the drug (2,5 and 5 mg/kg) reduced the response on days 7 and 11, respectively (*p <* 0.001; one-way ANOVA versus their respective db/db mice treated with vehicle).

### Effects of SFN treatment on the blood glucose levels and body weight

**Fig 2:**
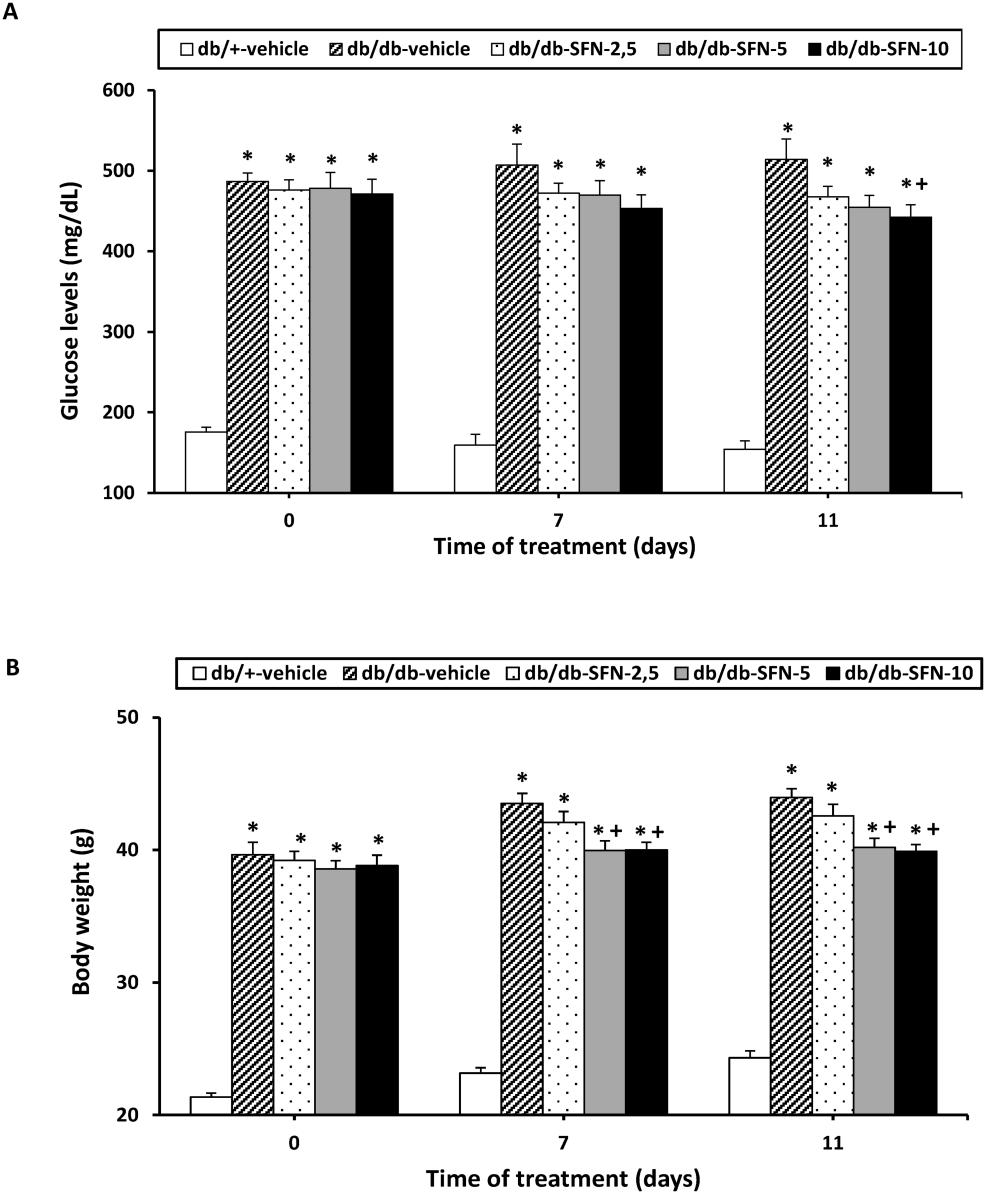
Effects of SFN treatment on blood glucose levels and body weight in db/db mice. The glucose levels (mg/dL) and body weight (g) in db/db mice treated with vehicle or SFN at 2.5, 5 and 10 mg/kg during 0, 7 and 11 consecutive days are shown. Glucose levels (A) and body weight (B) in db/+ mice treated with vehicle are also represented. For each parameter and day evaluated, * indicates significant differences as compared to db/+ mice treated with vehicle (*p <* 0.001 one-way ANOVA followed by the Student Newman Keuls test) and + indicates significant differences as compared to db/db mice treated with vehicle (*p <* 0.001 one-way ANOVA followed by the Student Newman Keuls test). Results are shown as mean values ± SEM; n = 6 animals per experimental group.

In this study, the effects of the intraperitoneal administration of different doses (2.5, 5 and 10 mg/kg) of SFN for 11 consecutive days on blood glucose levels and body weight of db/db mice were investigated. On days 0, 7 and 11 of treatment, blood glucose levels and body weight were measured. The two way ANOVA repeated measures revealed a significant effect of the treatment (*p <* 0.001) but not of the days of administration and their interaction. Indeed, although the increased blood glucose levels observed in db/db mice (*p <* 0.001; one-way ANOVA versus db/+ mice treated with vehicle) were significantly reduced by the administration of SFN at 10 mg/kg during 11 consecutive days (*p <* 0.001; one-way ANOVA versus db/db mice treated with vehicle; Fig 2A), SFN treatment at 2,5 and 5 mg/kg did not produce any significant effect on the glucose levels.

The effects of SFN treatment on body weight were also evaluated. The two way ANOVA repeated measures revealed a significant effect of the treatment (*p <* 0.001) and days of administration (*p <* 0.001) but not of their interaction. Indeed, a significant reduction in body weight of db/db mice treated with 5 and 10 mg/kg of SFN at 7 and 11 days of treatment was demonstrated (Fig 2B; *p <* 0.001; one-way ANOVA versus db/db mice treated with vehicle).

### Effects of the administration of DPDPE and SNC-80 on the mechanical allodynia

**Fig 3:**
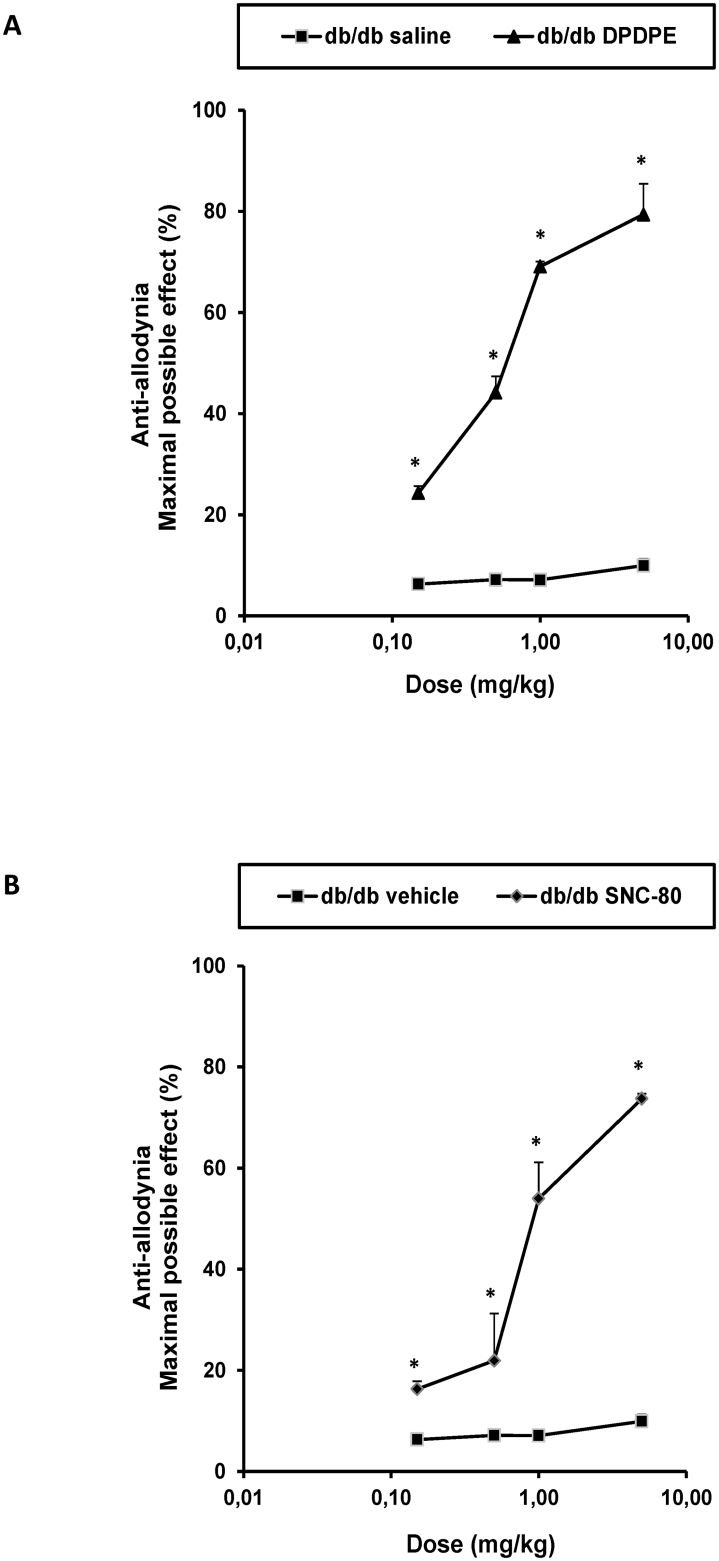
Effects of the subcutaneous administration of DPDPE or SNC-80 on the mechanical allodynia. Anti-allodynic effects of the subcutaneous administration of different doses (logarithmic axis) of DPDPE (A) or SNC-80 (B) and their respective controls in the hind paws of db/db mice are represented. Data are expressed as mean values of maximal possible effect (%) ± SEM (6 animals for dose). For each drug and dose, * indicates significant differences versus their respective db/db saline or vehicle treated mice (*p <* 0.05, one-way ANOVA followed by the Student Newman Keuls test). Results are shown as mean values ± SEM.

The subcutaneous administration of DPDPE (Fig 3A) or SNC-80 (Fig 3B) dose-dependently inhibited the mechanical allodynia in db/db mice. Indeed, the anti-allodynic effects produced by 0.15, 0.5, 1 and 5 mg/kg of DPDPE or SNC-80 were significantly higher than those produced in their corresponding db/db saline or vehicle treated animals (*p <* 0.001, one way ANOVA followed by the Student Newman Keuls test).

### Reversal of the antinociceptive effects of DPDPE and SNC-80 by naltrindole

The anti-allodynic effects produced by the subcutaneous administration of 5 mg/kg of DPDPE (Table 1) or SNC-80 (Table 2) in db/db mice were completely reversed by intraperitoneal administration of a selective DOR, naltrindole at 2 mg/kg (*p <* 0.001; one way ANOVA followed by Student Newman Keuls test). The intraperitoneal administration of naltrindole in db/db mice did not have any significant effect on the mechanical nociceptive response evaluated in this study.

**Table 1:** Effects of the subcutaneous administration of 5 mg/kg of DPDPE alone or combined with 2 mg/kg of naltrindole on the mechanical allodynia (von Frey filaments strength, g) in db/db mice.

*Treatment*	*Mechanical response von Frey filaments strength (g)*
saline-vehicle	1.7 ± 0.1
DPDPE -vehicle	3.0 ± 0.1*
DPDPE-naltrindole	1.7 ± 0.1
saline-naltrindole	1.8 ± 0.1

Results are shown as mean values ± SEM; n = 6 animals per experimental group.

* *p <* 0.05 denotes significant differences versus saline plus vehicle treated group (one way ANOVA, followed by the Student Newman Keuls test)

**Table 2:** Effects of the subcutaneous administration of 5 mg/kg of SNC-80 alone or combined with 2 mg/kg of naltrindole on the mechanical allodynia (von Frey filaments strength, g) in db/db mice.

*Treatment*	*Mechanical response von Frey filaments strength (g)*
vehicle-vehicle	1.7 ± 0.2
SNC-80 -vehicle	2.7 ± 0.1*
SNC-80-naltrindole	1.7 ± 0.2
vehicle-naltrindole	1.8 ± 0.1

Results are shown as mean values ± SEM; n = 6 animals per experimental group.

* *p* < 0.05 denotes significant differences versus vehicle plus vehicle treated group (one way ANOVA, followed by the Student Newman Keuls test).

### Effects of SFN treatment on the anti-allodynic effects of DPDPE and SNC-80

**Fig 4:**
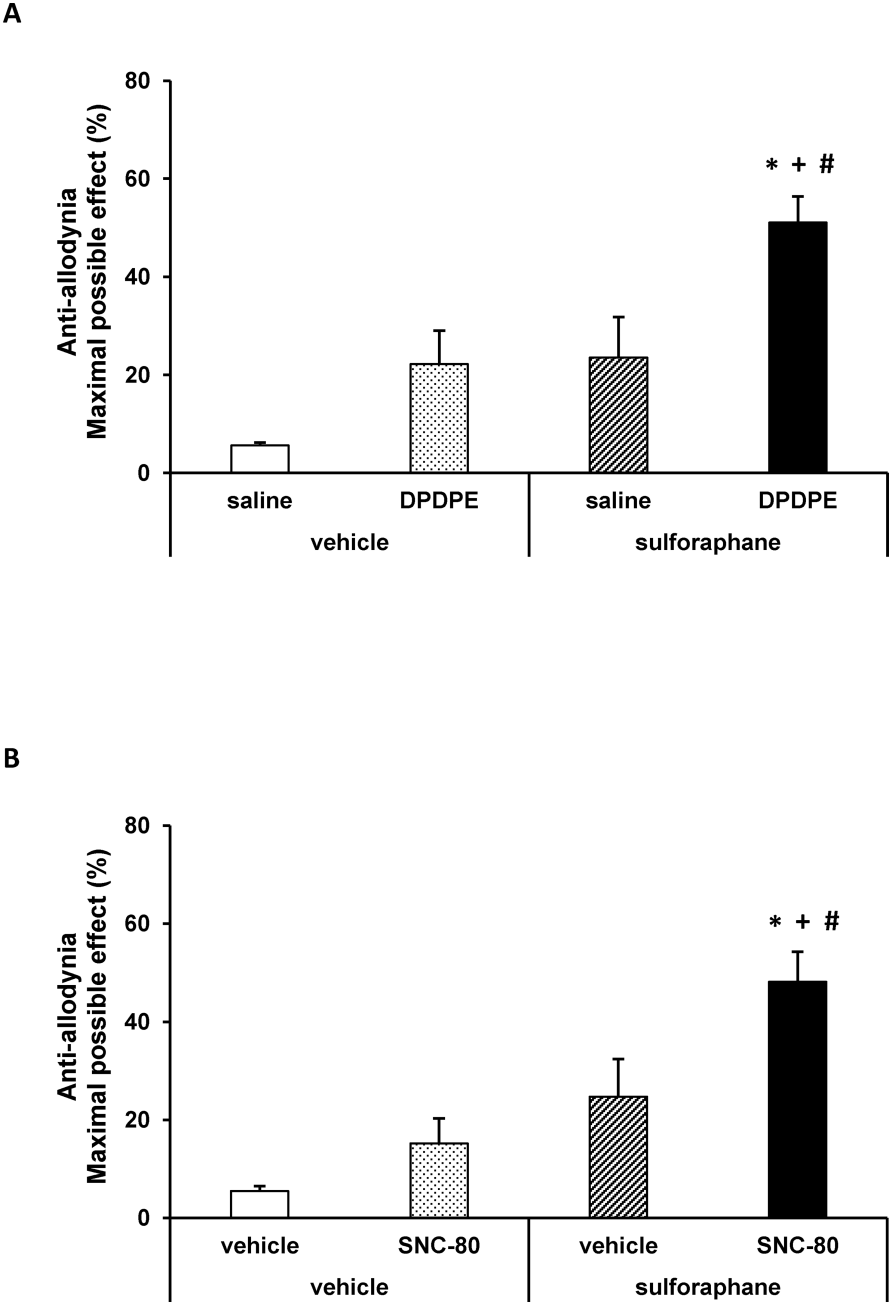
Effects of SFN on the anti-allodynic effects of DPDPE and SNC-80. Anti-allodynic effects of the subcutaneous administration of 0.15 mg/kg of DPDPE (A) or 0.5 mg/kg of SNC-80 or their respective vehicle in the hind paws of db/db mice pretreated with 10 mg/kg of SFN or vehicle are represented. The effects of the intraperitoneal administration of SFN alone are also shown. For each drug tested, * denotes significant differences versus mice treated with vehicle plus saline or vehicle (*p <* 0.05, one way ANOVA followed by Student Newman Keuls test), + denotes significant differences versus animals treated with vehicle plus drug (p<0.05, one way ANOVA followed by the Student Newman Keuls test) and # denotes significant differences versus animals treated with SFN plus saline or vehicle (*p <* 0.05; one way ANOVA followed by the Student Newman Keuls test). Data are expressed as mean values of the maximal possible effect (%) ± SEM; n = 6 animals per experimental group.

The effects of the intraperitoneal administration of 10 mg/kg of SFN or vehicle (DMSO 1%) on the anti-allodynic effects produced by the subcutaneous administration of low doses of DPDPE (0.15 mg/kg), SNC-80 (0.5 mg/kg), saline or vehicle in db/db mice were investigated. Our results showed that the administration of SFN combined with the subcutaneous administration of a low dose of DPDPE (Fig 4A) significantly enhanced the mechanical anti-allodynic effects produced by these drugs as compared to their respective control groups treated with vehicle plus saline or DPDPE as well as to animals treated with SFN plus saline (*p <* 0.01, one way ANOVA followed by the Student Newman Keuls test). Similar results were obtained regarding SNC-80 (Fig 4B). That is, the co-administration of SFN and SNC-80 significantly enhanced the mechanical anti-allodynic effects produced by this drug as compared to their respective control groups treated with vehicle plus vehicle or SNC-80 as well as to mice treated with SFN plus vehicle (*p <* 0.01, one way ANOVA followed by the Student Newman Keuls test).

### Effects of SFN treatment on Nrf2, HO-1, NQO1, JNK and DOR protein levels

**Fig 5:**
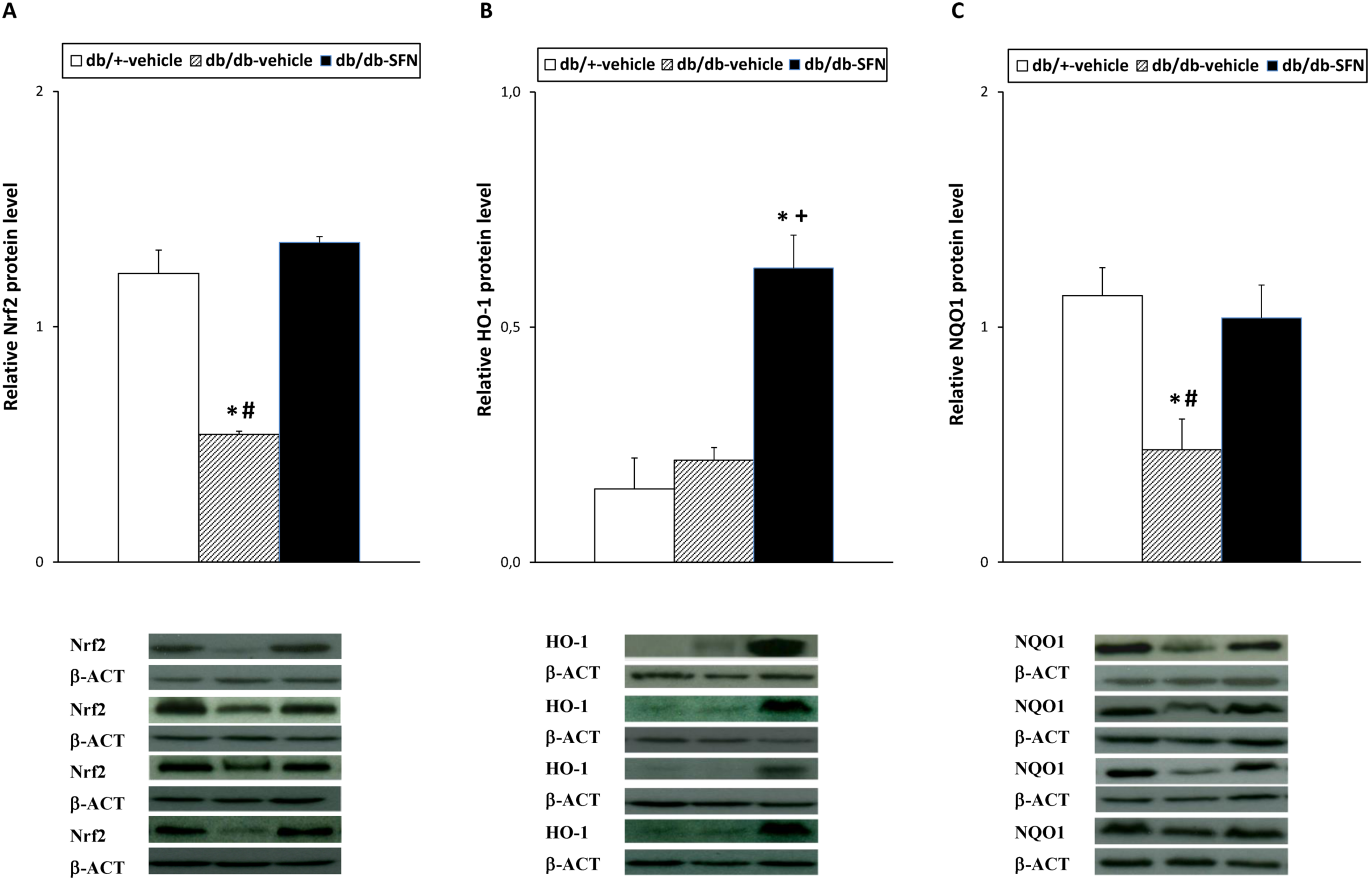
Effects of SFN treatment on the protein levels of Nrf2, HO-1 and NQO1 in the sciatic nerve from diabetic mice. The protein levels of Nrf2 (A), HO-1 (B) and NQO1 (C) from db/db mice treated with vehicle or SFN are represented. The expression of Nrf2, HO-1 and NQO1 from db/+ mice treated with vehicle has been also represented as controls. For each protein, * indicates significant differences when compared versus db/+ mice treated with vehicle (*p <* 0.05, one-way ANOVA followed by Student Newman Keuls test); # indicates significant differences when compared versus db/db mice treated with SFN (*p <* 0.05, one-way ANOVA followed by Student Newman Keuls test) and + indicates significant differences when compared versus db/db mice treated with vehicle (*p <* 0.05, one-way ANOVA followed by Student Newman Keuls test). Western blots images for Nrf2, HO-1 and NQO1 proteins in which β-actin was used as a loading control for 4 samples per group are also shown. Data are expressed as mean values ± SEM; n = 4 samples per group.

The protein levels of the transcription factor Nrf2, HO-1, NQO1, JNK and DOR in the sciatic nerve from db/db mice treated with 10 mg/kg of SFN or vehicle as well as from db/+ mice treated with vehicle are shown. Our results demonstrated that the reduced expression of Nrf2 observed in the sciatic nerve of diabetic mice (Fig 5A; *p <* 0.022; one-way ANOVA versus db/+ mice treated with vehicle) was reversed by the administration of SFN (*p <* 0.022; one-way ANOVA versus db/db mice treated with vehicle). Our results also shown that while the sciatic nerve protein levels of HO-1 were not altered in diabetic mice (Fig 5B), a significant increased expression of this enzyme was observed in the sciatic nerve from db/db mice treated with SFN (*p <* 0.03; one-way ANOVA versus db/+ and db/db mice treated with vehicle). Moreover, the decreased expression of NQO1 observed in the sciatic nerve from db/db mice (Fig 5C; *p <* 0.013; one-way ANOVA versus db/+ mice treated with vehicle) was completely reversed by SFN treatment.

**Fig 6:**
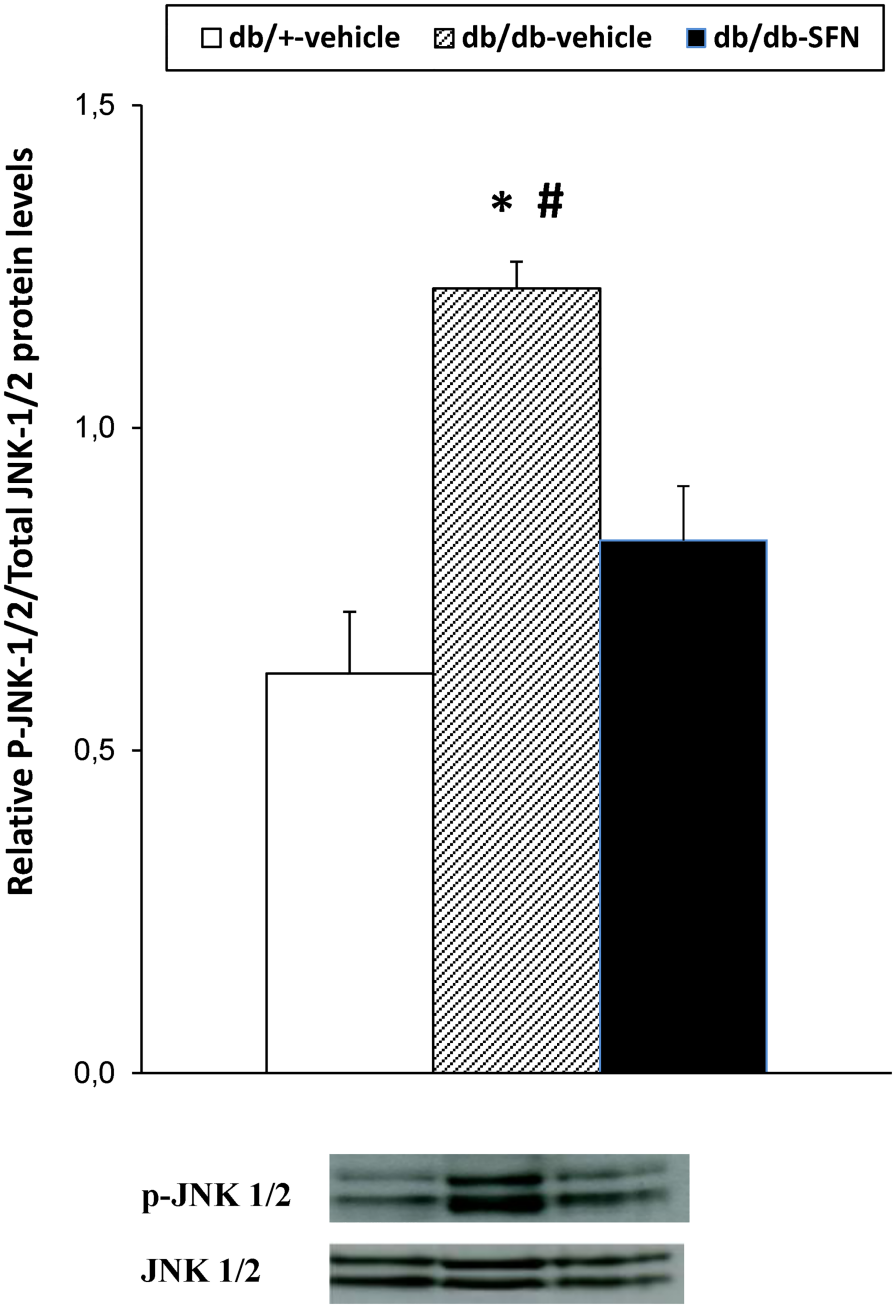
Effects of SFN treatment on the protein levels of JNK in the sciatic nerve from diabetic mice. Protein levels of JNK from db/db mice treated with vehicle or SFN are represented. The expression of JNK from db/+ mice treated with vehicle has been also represented as controls. * indicates significant differences when compared versus db/+ mice treated with vehicle (*p <* 0.05, one-way ANOVA followed by Student Newman Keuls test) and # indicates significant differences when compared versus db/db mice treated with SFN (*p <* 0.05, one-way ANOVA followed by Student Newman Keuls test). The density of phosphorylated JNK and total JNK was determined and the ratio was calculated and represented. Representative examples of western blots for phosphorylated JNK and total JNK proteins are also shown. Data are expressed as mean values ± SEM; n = 4 samples per group.

**Fig 7:**
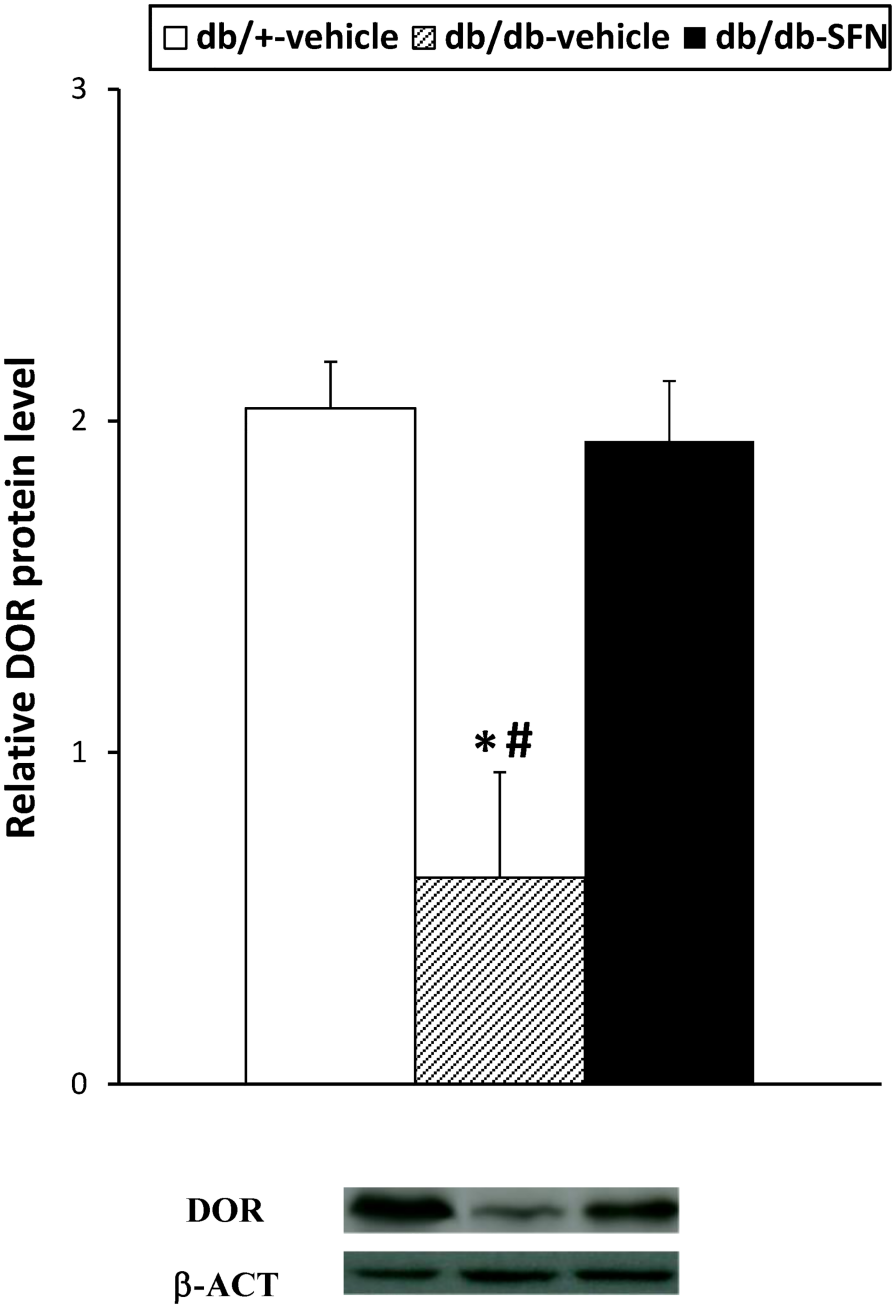
Effects of SFN treatment on the protein levels of DOR in the sciatic nerve from diabetic mice. Protein levels of DOR from db/db mice treated with vehicle or SFN are represented. The expression of DOR from db/+ mice treated with vehicle has been also shown as controls. * indicates significant differences when compared versus db/+ mice treated with vehicle (*p <* 0.05, one-way ANOVA followed by Student Newman Keuls test) and # indicates significant differences when compared versus db/db mice treated with SFN (*p <* 0.05, one-way ANOVA followed by Student Newman Keuls test). A representative example of western blot for DOR proteins, in which β-actin was used as a loading control, is also shown. Data are expressed as mean values ± SEM; n = 4 samples per group.

Our results also demonstrated that the increased phosphorylation of JNK observed in the sciatic nerves of db/db mice in comparison to db/+ mice treated with vehicle (Fig 6; *p <* 0.001; one-way ANOVA) was inhibited with SFN treatment. Finally, regarding DOR protein levels, our data revealed that SFN treatment also reversed the decreased expression of DOR in the sciatic nerve from diabetic mice (Fig 7; *p <* 0.025; one-way ANOVA versus db/db mice treated with vehicle).

## Discussion

In this study, we demonstrated that the intraperitoneal administration of SFN completely reversed the mechanical allodynia and reduced glucose levels and body weight gain in a mouse model of type 2 diabetes. In addition, the decreased sciatic nerve protein levels of Nrf2 and NQO1 associated to diabetes were reversed by SFN treatment, which also enhanced the expression of HO-1 and reduced the phosphorylation of JNK in the sciatic nerve of diabetic mice. The results of this study also indicated that the administration of each of the two DOR agonists, DPDPE and SNC-80, dose dependently inhibited the mechanical allodynia in db/db mice and treatment with SFN increased its anti-allodynic effects by normalizing the down regulation of DOR in the sciatic nerve of diabetic mice.

It is well known, that the activation of the Nrf2/HO-1 system increased insulin sensitivity and glucose tolerance in diabetic animals [29, 30]. Moreover the activation of this signaling pathway also suppressed body weight gain in the high-fat-diet-induced obesity in rodents and in obese *ob* mice [29, 31–34] revealing its anti-obesity effects. Our results supported these findings and further demonstrated that activation of Nrf2 by SFN reduced hyperglycemia and obesity associated with type 2 diabetes in db/db mice.

In the present work, we also demonstrated that the intraperitoneal administration of SFN inhibited the mechanical allodynia associated to type 2 diabetes in a dose dependent manner. These results are in accordance to the antinociceptive effects produced by this compound in rats with type 1 diabetes [28] and further revealed the effectiveness of this Nrf2 transcription factor activator in type 2 diabetic mice.

It is well known that the transcription factor Nrf2 is involved in the regulation of the antioxidant defense mechanisms by enhancing the production of endogenous antioxidant and detoxifying enzymes, such as HO-1 and NQO1, and decreasing the expression of several inflammatory mediators activated by MAPKs [28]. Therefore, in order to evaluate the possible mechanisms of the anti-allodynic effects produced by SFN in type 2 diabetic mice, we measured changes in the protein levels of Nrf2, HO-1, NQO1, and JNK in the sciatic nerve from SFN treated db/db mice. The decreased protein levels of Nrf2 observed in the sciatic nerve from db/db mice confirmed that the reduced expression of this transcription factor is associated to diabetes [28, 35]. This down-regulated expression of Nrf2 was consequence of hyperglycemia that contributes to prevailing oxidative conditions in peripheral nerves [6]. Our data also revealed that SFN treatment completely reversed the down regulated protein levels of Nrf2 in the sciatic nerve from db/db mice, indicating the participation of this transcription factor in the anti-allodynic effects produced by SFN on type 2 diabetic mice. Moreover, protein levels of the antioxidant enzyme HO-1 were significantly increased in the sciatic nerve from db/db mice treated with SFN. In agreement with our data, an increased expression of HO-1 in several tissues from type 1 diabetic animals treated with different Nrf2 or HO-1 inducers compounds has been also previously reported [14, 28, 36, 37]. Tacking account the corroborated antinociceptive properties produced by the activation of HO-1 under inflammatory and neuropathic pain conditions [8, 11, 14], our results suggested that the improvement of the mechanical allodynia induced by SFN in db/db treated mice might be also mediated by enhancing the HO-1 expression in the sciatic nerve of these animals.

It is well known that NQO1 is a highly inducible enzyme also regulated by the Nrf2 transcription factor and implicated in the protection against oxidative stress. Indeed, NQO1 knockout diabetic mice had increased hyperglycemia and higher ROS expression, revealing the important neuroprotective role played by this enzyme during diabetes [38, 39]. As expected, we demonstrated a reduced expression of this enzyme in the sciatic nerve of db/db mice as occurs in type 1 diabetic animals [28]. More interesting is the fact that SFN treatment normalized the down-regulated expression of NQO1 in db/db mice, revealing that the anti-allodynic effects produced by this compound in our diabetic mice might be also in part mediated by the protection against oxidative stress induced by NQO1 in diabetic animals.

Several works demonstrated that a downstream mechanism of inflammation in diabetic neuropathy include the activation of the JNK pathway [15, 40]. Our results supported these findings by demonstrating its activation in the sciatic nerve of db/db mice and further revealed that the induction of Nrf2 also has an inhibitory effect on JNK activation. Since JNK mediated multiple inflammatory mediators in type 2 diabetes, a reduction in its activity may be also involved in the antiallodynic effects of SFN in db/db mice. In summary, our results indicated that the antinociceptive produced by SFN in db/db mice might be mediated by the activation of Nrf2 signaling and the consequent reduction in blood glucose levels and JNK phosphorylation in the sciatic nerve of these animals.

In this study, we also evaluated the role played by DOR on the modulation of painful diabetic neuropathy. Our data revealed that the subcutaneous administration of two specific DOR agonists, DPDPE and SNC-80, inhibited mechanical allodynia in a dose dependent manner. These results are consistent with previous studies, in which the central, spinal or systemic administration of DPDPE dose-dependently inhibited the mechanical and thermal hypersensitivity manifested in type 1 diabetic animals [20, 22, 41]. Our results supported these findings and further revealed the potential use of DOR agonists for the treatment of painful diabetic neuropathy associated to type 2 diabetes. The specificity of the anti-allodynic effects produced by DPDPE and SNC-80 was demonstrated by the complete reversion of its effects with their co-administration with naltrindole, a selective DOR antagonist. The present study also demonstrated for the first time, that the administration of SFN significantly enhanced the anti-allodynic effects produced by low doses of DPDPE and SNC-80 in type 2 diabetic mice. These findings indicated that the antinociceptive effects produced by DOR agonists in type 2 diabetes might be modulated by the Nrf2 transcription factor activation. Our data also demonstrated that treatment with SFN avoided the sciatic nerve down regulation of DOR in db/db mice, indicating that the normalized peripheral expression of DOR induced by this Nrf2 transcription factor activator might explained the enhanced anti-allodynic effects produced by DPDPE and SNC-80 in SFN treated animals. Accordingly, a recent work has demonstrated reduced mRNA expression of DOR in the spinal cord of type 2 diabetic monkeys [42]. Nevertheless, the fact that SFN treatment also activated the expression of HO-1 in db/db mice supported the idea that the improvement of the anti-allodynic actions of DOR agonists produced by SFN might be also consequence of the induction of HO-1, as occurs in animals with peripheral inflammation or type 1 diabetes [12, 22]. Nonetheless, the normalization of the sciatic nerve NQO1 proteins and the inhibition of JNK phosphorylation induced by the Nrf2 inductor might also contribute to enhance the anti-allodynic effects of DOR agonists in SFN treated mice.

In conclusion, this study reports for first time that SFN treatment reverses mechanical allodynia and enhances the antinociceptive effects of DOR agonists by normalizing Nrf2, NQO1 and DOR down-regulation, increasing HO-1 expression, reducing hyperglycemia and inhibiting JNK phosphorylation in the sciatic nerve of diabetic mice. These data propose the administration of SFN alone and/or combined with DOR agonists as interesting approaches for the management of painful neuropathy associated to type 2 diabetes in mice.
